# Participant perspective on the recall-by-genotype research approach: a mixed-method embedded study with participants of the CHRIS study

**DOI:** 10.1038/s41431-022-01277-6

**Published:** 2023-01-04

**Authors:** Roberta Biasiotto, Maria Kösters, Katharina Tschigg, Peter P. Pramstaller, Norbert Brüggemann, Max Borsche, Christine Klein, Andrew A. Hicks, Deborah Mascalzoni

**Affiliations:** 1 Department of Biomedical, Metabolic and Neural Sciences, University of Modena and Reggio Emilia, Modena, Italy; 2 Institute for Biomedicine, Eurac Research, Affiliated Institute of the University of Lübeck, Bolzano, Italy; 3 Department of Cellular, Computational, and Integrative Biology, University of Trento, Trento, Italy; 4 Institute of Neurogenetics, University of Lübeck, Lübeck, Germany; 5 Department of Neurology, University Medical Center Schleswig-Holstein, Campus Lübeck, Lübeck, Germany; 6 Centre for Research Ethics and Bioethics, Department of Public Health and Caring Sciences, Uppsala University, Uppsala, Sweden

**Keywords:** Genetics research, Ethics

## Abstract

Recall-by-genotype (RbG) research recruits participants previously involved in genetic research based on their genotype. RbG enables the further study of a particular variant of interest, but in recalling participants, it risks disclosing potentially unwanted or distressing genetic information. Any RbG strategy must therefore be done in a manner that addresses the potential ethical and social issues. As part of an RbG pilot on the penetrance of Parkinson’s disease variants, we conducted an empirical mixed-method study with 51 participants of the Cooperative Health Research in South Tyrol (CHRIS) study to understand participant views on RbG research approach. Participants were disclosed the disease under investigation but not the individual variant carrier status. Results showed that participants filtered the information received through personal experience and enacted mechanisms to address the concerns raised by invitation by resorting to personal resources and the support provided by experts. While the non-disclosure of the *Parkin* variant carrier status was deemed acceptable, disclosing the disease under study was important for participants. Participant preferences for disclosure of the disease under investigation and the carrier status varied according to how the knowledge of individual carrier status was perceived to impact the participant’s life. This study provided insights into participant response to the RbG research approach, which are relevant for RbG policy development. A suitable communication strategy and granular options addressing preferences for invitation in the original informed consent are critical for an ethically informed RbG policy.

## Introduction

A recall-by-genotype (RbG) research approach consists of inviting individuals who previously participated in genetic research based on their specific genotypes. The aim is to efficiently study the phenotype of those carrying specific variants of interest [1].

This research approach, which is becoming increasingly popular thanks to the large availability of genotyping data from the most recent sequencing technologies, poses legal and ethical challenges. Inviting participants and providing information on the research study may imply disclosing genetic information, disease risk, and unwanted or distressing information [2–6]. For this reason, exploring participant perspectives on the RbG approach is crucial to improve research practices and develop suitable policy. The Cooperative Health Research in South Tyrol (CHRIS) study, a longitudinal study with the general population of Val Venosta/Vinschgau, South Tyrol in Italy, conducted its first RbG study in a sub-sample of the CHRIS cohort (pilot RbG study) as a joint project with the University of Lübeck, in the framework of the ProtectMove Research Unit project (FOR2488), in 2018 [7, 8]. The ProtectMove project investigates movement disorders, including Parkinson’s disease. The pilot RbG study aimed to investigate the phenotypic features of carriers of heterozygous *Parkin* variants. The variants under study, carried in a heterozygous state, had low penetrance, leading to mild or no neurological symptoms.

Our study, a mixed-method empirical study embedded within the pilot RbG study, aims to understand participant perspectives on RbG research approach and participant experience of invitation and participation in a specific RbG study. The present work was motivated by process of policy design for RbG studies within the CHRIS study [7]. A participant-centered approach, which considers participant views, is key in the CHRIS study, which uses dynamic consent as an informed consent model [9–11].

## Methods

### Study setting

The CHRIS study focuses on age-related neurological, cardiovascular, and metabolic health and diseases and their genetic, environmental, and lifestyle determinants [9]. The study, which started in 2011, collects data and biological samples from a closed cohort of 13,389 adult participants enrolled between 2011 and 2018. The mother tongue of most residents in Val Venosta/Vinschgau is German [11]. Participant recruitment and the broader empirical RbG study have been described elsewhere [7, 8] and summarized in Table 1.

**Table 1:** Pilot RbG study and recruitment of participants.

Project	The ProtectMove Research Unit project, focused on Parkinson’s disease and other neurological movement disorders, aims to investigate the genetic penetrance of specific variants of many genes, including the *Parkin* gene.
Pilot	In order to guarantee participants’ safety, the RbG was conceived as a pilot study focused on heterozygous *Parkin* variants that are pathogenic when inherited recessively in a homozygous or compound heterozygous state, but may influence symptoms dominantly, with very low penetrance, in a heterozygous state. Carriers of heterozygous *Parkin* variants may show subtle signs of Parkinson’s disease, slight abnormalities in the dopaminergic system [26, 27], morphometric changes in the basal ganglia [28], and compensatory changes in functional MRI studies [29]. The pilot study consisted of deeper neurological examinations, transcranial ultrasounds, and quantitative movements assessment to explore the genotype-phenotype relationships in heterozygous carriers versus non-carriers.
Design	The study was designed with a matched recruitment, where half of the invited participants carry the *Parkin* variant of interest in a heterozygous state, and the other half, who do not carry the variant, serve as controls. Controls were chosen as closely related to the carriers, with similar age and same sex, if possible.
Invitation	Participants were invited through the mail, which included a letter and information on the study.
Information provided in the invitation	The invitation explained the aim of the study, disclosed the disease and the variants under study, and the study’s design. Participants were informed that, according to current knowledge, heterozygosity of the *Parkin* variant under study does not cause Parkinson’s disease, but may cause an increase of the risk of prodromal neurological symptoms. As being a carrier of the variant in a heterozygotic state is not associated with any known clinical benefit, participants were informed that individual carrier status would not be disclosed.
Disclosure	Both researchers and participants did not know the participant’s individual carrier status (double blind).
Further communication	Invited participants were provided a phone number to call to ask clarifications and questions, if needed, and received a phone call from the study assistants to fix an appointment for the clinical examination and the empirical study.

A total of 58 participants were invited to the pilot RbG study, with the strategy described in the table.

### Mixed method rationale

The research was designed with a mixed-method approach with a convergent design [12, 13].

Quantitative and qualitative methods were used as a complementary strategy.

Data analysis and results interpretation were conducted as follows:

(1) Quantitative and qualitative data were collected through two questionnaires and a semi-structured interview. The questionnaire and the interview guide were similar in their general structure and content to offer participants both written and verbal tools to express opinions and share emotions (Supplementary Information 1).

(2) Quantitative and qualitative data were first analyzed separately with suitable methods.

(3) This was followed by an integrated interpretation of the results: The quantitative results were interpreted and incorporated ad hoc within the themes found with the thematic analysis.

### Data collection

Data collection occurred in Schlanders/Silandro (Italy) at the CHRIS study center on August 20–24, 2018. The workflow was as follows: (1) First questionnaire; (2) Clinical examination conducted by medical doctors; (3) Interview; (4) Second questionnaire. DM and MK jointly conducted the interviews in German and collected fieldnotes. The approximate duration of the interview was 20 min. Due to time constraints in the daily workflow, respondents sometimes answered the second questionnaire before the interview. In four cases, the second questionnaire was filled in together with the interviewers, which helped respondents go through the questions.

### Data analysis

#### Quantitative data

Descriptive statistics of the quantitative data were generated using Microsoft Excel.

#### Qualitative data

The interviews were recorded and transcribed verbatim by a professional transcription service. The German transcripts were translated into the English language using Deepl Pro Advanced. Transcripts were analyzed using thematic analysis according to the six-step process described by Braun and Clarke [14]. Our approach was grounded in critical realism/contextualism, i.e., reality is interpreted through participant experiences within broader social context [15, 16]. Coding and theme development were with an inductive approach. First, RB became familiar with the interview content by repeatedly reading the transcripts and annotating memos. Second, with an iterative approach and an ongoing coding development and refinement process, the author categorized the data by developing initial codes. Fieldnotes collected by the authors who conducted the interviews and memos generated during the data familiarization were helpful in this process. Third, the codes were grouped through an abstraction process to generate broader concepts. Candidate themes and sub-themes, and their relationship as well, were developed. In steps 4 and 5, themes were reviewed, then defined and finalized. Lastly, findings were reported in an analytic narrative. Exemplary quotes were included ad hoc to support the interpretation (in Results). The exemplary quotes are indicated with an “I” followed by a number to identify each individual interview. Findings were situated within the existing relevant literature (in Discussion).

ATLAS.ti 8 and Microsoft Excel were used as data management and analysis software.

### Validation of translation

To validate the translation, KT, a German mother tongue, verified the correspondence of the English translation with the original texts in German. As a further step, MK, a German mother tongue, and KT independently coded part of the German transcripts using ATLAS.ti 8. RB compared, assessed, and confirmed the overall consistency of the coding in English and German.

## Results

A total of 50 CHRIS participants invited to participate in the study agreed to participate (100% of the RbG study participants). A relative of one of the invitees asked to join and was included in the empirical study. One participant left the study center without participating in the interview. As a whole, 51 participants responded to the questionnaires, and 50 participated in the interview. Table 2 shows the socio-demographic characteristics.

**Table 2:** Socio-demographic description of participants.

	*N*
Age range (years)	
25–34	12
35–44	8
45–54	12
55–64	13
65–74	2
>75	4
Education	
Primary school	1
Lower secondary school	5
Vocational school	22
Upper secondary school	13
University or higher	10
Total	51

Participants included 24 females and 27 males. Participants’ age was in the range of 25–80 years. Female respondents’ median age was 48 years, and the age range was 31–80 years. For male respondents, the median age was 47 years, and the age range was 25–75 years.

We identified four main themes. They represent patterns of meaning concerning participant response to RbG invitation and participation. Participant response was thematized as follows:

- Information filter through personal experience: Participants filtered received information through personal experience. This occurred through reflecting on heredity and the reaction to receiving the invitation.
- Stress relief mechanisms: Participants enacted mechanisms to alleviate stress related to the invitation and participation in an RbG study. They consisted in resorting to personal resources and in seeking support from experts.
- Targeted information matters: Targeted information was important for participation. Participants decided based on the information received, and participant engagement depended on the information received.
- Expectations on disclosure: The expectation concept included what is desirable (or non-desirable) and acceptable (or non-acceptable) to know or not to know in an RbG study scenario. Participant preferences for disclosure of the disease under study and of the carrier status varied according to how the knowledge of individual carrier status was perceived to impact participant’s life (Table 3).

**Table 3:** How the knowledge of individual carrier status is perceived to impact a participant’s life determines preferences for disclosure.

		How knowledge of the individual carrier status is perceived to impact participant’s life		
Type of variant	Variant that does not cause a disease	Knowledge allows awareness		Knowledge is - Irrelevant - Unnecessary burden
	Variant that causes a disease	Knowledge allows - Possible action (prevention and/or treatment) - Coping	Knowledge allows action (prevention and/or treatment)	Knowledge is a burden
		↓	↓	↓
Participant preference for disclosure of carrier status		Disclosure	Conditional disclosure	No disclosure

### Information filter through personal experience

The qualitative data analysis showed that, while reasoning on their recruitment and interpreting the invitation, participants resorted to their own experience and concept of genetic disease transmission. They inferred risk and carrier status from the disease’s familial history and other family members’ invitations. For example, inviting more people from the same family made a few participants presume that the whole family was a carrier of the genetic variant or was affected by the disease:

“When I got the invitation, my mother got the invitation at the same time, and my two half-siblings didn’t. That made me think a bit at first, because I thought, okay, it could also be that I got this gene from my mother and from my biological father and, as my two siblings have a different father, didn’t get the gene, because they probably don’t carry it or don’t carry it in duplicate. That was my first train of thought. Then my mother’s sister and her son were also invited. So, I thought, maybe it’s something that really affects the whole family because four people from one family is quite a lot. I47”

The invitation made participants think about Parkinson’s disease and the risk of developing the disease. They reflected on their health, speculated on their carrier status, and the possibility of genetic transmission of the variant to future generations. In the questionnaire, more than half of the participants thought about the possibility of being carriers of the *Parkin* gene variant (Table 4, A1). Participants expressed different degrees of concern related to being a carrier of the variant and of the perceived likelihood of belonging to the control group (A2, A3). Participation in the RbG study caused an increase in worries and risk perception of developing Parkinson’s disease only in a small fraction of participants (A4, A5). Additionally, data showed that participants struggled with estimating the risk of developing the disease (A6: 41% chose I don’t know). 35% of participants reported a family history of Parkinson’s disease (A7).

**Table 4:** Results of the survey.

Answer n. (An) (Qn, qn)	Topic of the question	Results			Interpreted within the theme
A1 (Q2, q2)	After the invitation, thought about being a carrier of the variant associated with Parkinson’s disease	Yes	27	52.9	Information filter through personal experience
		No	21	41.2	
		Not answered	3	5.9	
A2 (Q3, q2)	Concern about being carrier of the Parkin gene variant	Very concerned	1	2	
		Concerned to some extent	11	21.6	
		A little concerned	17	33.3	
		Not at all concerned	17	33.3	
		I don’t know	3	5.9	
		Not answered	2	3.9	
A3 (Q7, q2)	Perceived likelihood of not carrying the variant	Very likely	9	17.7	
		Rather likely	3	5.9	
		Neither likely nor unlikely	19	37.3	
		Rather unlikely	14	27.5	
		Very unlikely	5	9.8	
		Not answered	1	2	
A4 (Q5a, q2)	Comparative risk perception of developing Parkinson’s disease following invitation (risk perception higher today compared to risk perception before receiving the invitation)	Do not agree	20	39.2	
		Rather not agree	6	11.8	
		Neither agree nor disagree	17	33.3	
		Rather agree	5	9.8	
		Agree	2	3.9	
		Not answered	1	2	
A5 (Q5b, q2)	Comparative assessment of feeling worried about developing Parkinson’s disease following invitation: feeling more worried today compared to before receiving the invitation	Do not agree	21	41.2	
		Rather not agree	6	11.8	
		Neither agree nor disagree	15	29.4	
		Rather agree	7	13.7	
		Agree	0	0	
		Not answered	2	3.9	
A6 (Q4, q2)	Self-assessment of the risk of developing Parkinson’s disease	Very high risk	1	2	
		Increased risk	5	9.8	
		Low risk	18	35.3	
		No risk	5	9.8	
		I don’t know	21	41.2	
		Not answered	1	2	
A7 (Q8, q2)	Familial history of Parkinson’s disease	Yes	18	35.3	
		No	31	60.8	
		Not answered	2	2.9	
A8 (Q1, q1)	Satisfaction with information received before participation	No, many questions were not answered	2	3.9	Stress relief mechanisms
		No, some questions are still unclear	3	5.9	
		Yes, most of the questions were clarified	22	43.1	
		Yes, all the questions were answered	20	39.2	
		Not answered	4	7.8	
A9 (Q10, q2)	Satisfaction with information received at the study center	No, many questions were not answered	0	0	
		No, some questions are still unclear	0	0	
		Yes, most of the questions were clarified	14	27.5	
		Yes, all the questions were answered	36	70.6	
		Not answered	1	2	
A10 (Q11, q2)	Clarification of questions and doubts during participation	Yes	49	96.1	
		No	1	2	
		Not answered	1	2	
A11 (Q13b, q2)	Disclosure of the disease under study	Yes	45	88.2	Targeted information matters
		No	3	5.9	
		This is not important to me	0	0	
		Not answered	3	5.9	
A12 (Q9, q2)	No return of genetic research results	That’s fine with me	42	82.4	Expectations on disclosure
		I think that’s a pity	8	15.7	
		I don’t care	0	0	
		Not answered	1	2	
A13 (Q6, q2)	Willingness to know the individual carrier status relative to *Parkin*	Yes	27	52.9	
		No	23	45.1	
		Not answered	1	2	
A14 (Q13a, q2)	Evaluation of the practice of not disclosing the disease	Very negative	8	15.7	
		Rather negative	17	33.3	
		Partly negative/partly positive	16	31.4	
		Rather positive	8	15.7	
		Very positive	1	2	
		Not answered	1	2	
A15 (Q13c, q2)	Impact of the type of disease in willingness to know the disease under study	Yes	27	52.9	
		No	23	45.1	
		Not answered	1	2	

For easy comparison with the original questions of the questionnaires (Supplementary Information 1), question number (Qn) and questionnaire number (qn) are also reported.

### Stress relief mechanisms

In the interviews, a few participants observed that the invitation raised concerns and often temporary worries. These were solved by resorting to personal resources and the experts’ support. Personal resources consisted in re-reading the letter several times and consulting family members. Support from experts consisted of face-to-face conversations with the medical doctors conducting the clinical examination and interactions with the study assistants, medical doctors, and researchers involved in the study. Participation at the study center was perceived as a positive and comfortable experience. Consulting the experts provided relief, reassurance, and an opportunity for further clarification:

“I had to read the form twice, because the first moment I saw it, my first thought was: do I have Parkinson’s disease or something? […] Then, you don’t read through very carefully […] you read it with fear and maybe you only read out what you are already afraid of. And then I gave it to my husband to read and he read it more thoroughly and then I read it again myself and then I really understood that it was actually, as the doctor explained it to me today. And the doctor’s explanation again today made it clear to me what it’s all about. […] it was very helpful to get rid of fear and also to know something about how this research works and proceeds. I16”

**Fig. 1:**
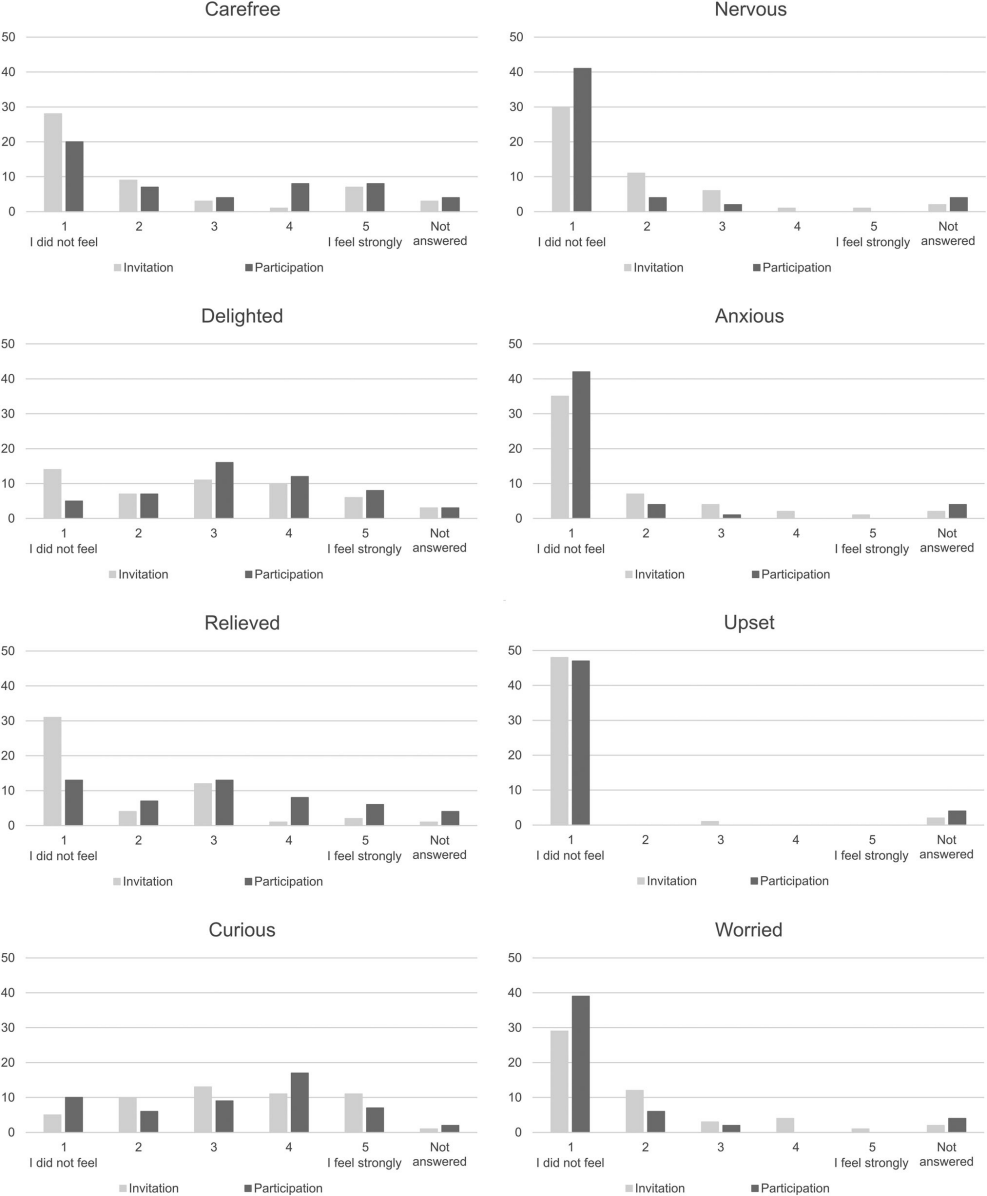
Participants’ emotions. Emotions at the time of receiving the invitation and after participation are shown in light gray and dark gray, respectively. On the y-axis, the number of participants reporting the indicated score (1 to 5) is shown.

A trend in emotions before and after participation was found through the quantitative data analysis. By comparing the distribution of the scores assigned in the questionnaire to each emotion at the time of the invitation and after participating in the study, the number of participants reporting positive emotions (carefree, delighted, relieved) with high intensity increased after participation, while the number of participants reporting negative emotions (nervous, anxious, worried) with high intensity decreased after participation (Fig. 1). For the “curious” emotion, a specific pattern on the score assigned was not identified. Participants reported not feeling upset when receiving the invitation or after participating (except one). Additionally, none of the respondents regretted being invited to the study (50 did not prefer not to be invited, and one not answered).

A similar positively correlated trend was found in the satisfaction with the information received before and after the visit: before coming to the study center, only a minority of the participants had questions unanswered or unclear; after participation, almost all respondents found that most or all their questions were addressed and answered, and had their doubts adequately clarified and addressed (Table 4, A8, A9, A10). In the interviews, participants raised issues of clarity, complexity, exhaustivity, and amount of the material provided as elements that affected their understanding of the information received in the invitation.

### Targeted information matters

Qualitative data showed that participants saw participation in the Parkin RbG study as a contribution to a meaningful cause with benefits for future generations and society. Personal interest in the clinical examinations and utility for own health were also reasons, shared by most participants, for participation:

“I can help research with it *[through participation in the study, Ed]*, but it also helps me, because I really do get a health check-up for free. I23”

“I found it interesting that something is being researched. Because I want to contribute to the results and if you can help, then why not. […] I thought that this could also be of use to me. If something turns out or if there are interesting things in the future. I15”

Additionally, motivations for participation were contextualized in terms of engagement with the CHRIS study:

“If you get an invitation, then it’s somehow an obligation to go. I46”

Quantitative data showed that most participants would like to know the disease associated with the gene variant under study (Table 4, A11). The analysis of qualitative data showed that, while non-disclosing the disease under study would negatively affect the willingness to participate and engagement, the interest for the specific disease under investigation worked as a motivator for participation:

“I am a nurse and have therefore often had to deal with Parkinson’s patients in my life. My grandmother had Parkinson’s disease. And a very good friend of mine had a very early form of Parkinson’s when she was just over 40. And I personally would be happy to support any study on this subject, like many other studies, if I could. I35”

A few participants clarified that being informed on the area of investigation and the aim of the study was important, not only to be aware of what they were contributing to but also not to feel objectified and instrumentalized as research participants:

“From my point of view, it is important to inform participants, considering that they participate as volunteers, otherwise, someone will be treated as a guinea pig or something. Blood is taken and one doesn’t know where and for what, and I would be in favor of the information simply being available. I14”

On the other hand, for a few respondents, the decision on participation was not based on the disease under study and disease disclosure.

### Expectations on disclosure

The survey showed that most participants were fine with genetic research results not being returned to them in either individual or aggregated form (Table 4, A12). Correspondingly, interview respondents deemed the practice of no return of genetic research results (either individual or aggregated results) acceptable. Participants expressed the view that genetic research results had no direct value for them and that they served for research purposes only:

“I am not interested in the findings for research purposes […] I am not a researcher. I49.”

Participants distinguished genetic research results from clinically relevant or actionable findings, which were expected to be returned, given their possible utility for their and their family’s health:

“For me, it is simply important that if I am really a risk patient, and that is clear from the results, that there is something coming up for me, and above all for my family, that I am perhaps informed. So, basically about genetic information, I can’t do anything with that. I13.”

Genetic research results were perceived to affect family also in a negative way:

“I think if I knew that I had some kind of genetic change that would lead to some kind of serious illness or something, then I would maybe decide not to have any more children […] Then I would be afraid. So, it would really be a drastic life change if you knew that. Then I would probably spend ages finding out whether I should have another child or not, or it would make me feel insecure. I18”

The quantitative data showed that respondents were split into two approximately equal groups as regards willingness to know their individual carrier status with respect to the *Parkin* variant under investigation (Table 4, A13). However, during the interviews, the majority of participants deemed the non-disclosure of the *Parkin* gene carrier status acceptable. Given that heterozygosity does not lead to the development of Parkinson’s disease, disclosing the carrier status was considered irrelevant, useless, and without diagnostic or predictive value for a few participants. A few participants not only found the non-disclosure acceptable but also did not want to know their carrier status because they perceived that such information would cause unnecessary burdens and concerns (e.g., being aware of one’s heterozygous condition may affect family planning). On the other hand, a few participants would have liked their carrier status to be disclosed: In this case, their motivation was curiosity or/and they found that such information contributed to their awareness about their health:

“I think I would worry, I don’t know, but I think I would listen to myself more purposefully or pay more attention to changes. […] I don’t think I would be more afraid, but I think that on the one hand, I would pay more attention and perhaps go to a doctor earlier, which could also be helpful. I23”

Quantitative data showed that almost all participants were willing to participate in further RbG studies (94.12% yes, 1.96% no, 3.92% not answered). Almost half of the participants evaluated not disclosing the disease under study in RbG studies negatively. In contrast, 17.65% in a positive way, and 31.37% found it partly positive and partly negative (Table 4, A14). For more than half of the participants, the type of disease would impact their decision about being willing to know the disease under study (A15).

During the interviews, participants were challenged with a hypothetical scenario in which an RbG study on a variant that increases the risk for a serious disease was conducted. They were explained that in such a case, they would have their carrier status disclosed and were asked to state their preference for being recontacted or not for participation and to explain their views. When provided scenarios, a variety of preferences were returned. Participants who wanted to know their carrier status saw its disclosure as a positive opportunity for prevention and actionability. This information was also seen to provide an awareness of susceptibility, which is essential for the development of responses and coping processes:

“I would like to know, you can prepare yourself somehow. You also have to protect the family somehow, so I would like to know. I13”

“If you have cancer or something, and there is perhaps the possibility that you can treat it at an early stage, it might be better to know at an early stage or what alternatives there might be. […] you can then deal with it a little or talk to people who are in the same situation and exchange ideas, on how are you doing in general, what are you limited in or what keeps you busy or do you somehow need more time for yourself, if you have to somehow cope with your fate now?. I29”

Actionability was used as a criterion for disclosure by those participants who wanted to be invited and to know their carrier status only if opportunities for prevention or therapy were available or in studies about non-causative variants:

“If there is a therapy, […] then I would like to know. If it’s something that you can’t do anything about at the moment anyway, then I personally would rather not know. I28”

A minority of participants did not want to know their individual carrier status nor wanted to be invited to participate in further studies on a pathogenic variant. They perceived that knowing the carrier status of a pathogenic variant was a burden:

“No, I don’t want to know. […] Because then it’s always in the back of my mind. I think that with every decision and with everything you have that in the back of your mind. I32”

Table 3 summarizes the above-described preferences.

## Discussion

The invitation is the first contact in the recruitment process of an RbG study. We found that the information in the invitation was interpreted within a framework of existing knowledge and assumptions of genetic risk and sometimes raised concerns amongst potential participants. Resorting to own resources to understand the role in research and the eligibility for RbG was also acknowledged in another study with biobank participants and cystic fibrosis patients [17]. After participation in the RbG study, the negative emotions tended to be primarily dissipated, and participants generally felt more at ease. This can be understood within the relief effect provided by the conversation with the medical doctors. These findings showed that information and support could impact emotions connected to RbG research approaches.

From this study, we observed that: (1) The emotional response that this kind of study may trigger should be considered, and communication strategies that minimize potential distress should be developed. (2) Considering that respondents reported that the amount of material might be a limit, there is the risk that long and detailed letters may be just skimmed instead of being read thoroughly. Therefore, an apt balance between complexity and clarity and between detailed information and an acceptable amount of material should be found. (3) Targeted, transparent and effective communication should be tailored to the variant under study.

Possible practical strategies are: (1) Provide layered information with increasing length and depth. (2) Provide visual tools that allow an adequate understanding of the contents, such as infographics, schemes, and boxes with key concepts. (3) Provide a short glossary with terms that are likely difficult to understand. (4) Identify appropriate lay language to explain research designs, the concept of carrier status, and the risk associated with carrying the variants under study. (5) Provide a contact reference to guarantee the possibility of asking questions and clarifications.

Furthermore, tailoring the communication to the variant under study means that, under certain conditions, it may be necessary to involve medical professionals and genetic consultants from the very first contact. Therefore, it is important to reflect not only on the content and amount of the information provided but also on the timing of different types of communication: which types of communication (written or oral) should come first, in which situations, and how and who should deliver information.

In this study, we found that information affected decision-making on participation and engagement. The disclosure of the disease under study was perceived as part of the transparency-based reciprocity relation between the participant and researcher. As motivators for participation, contribution to the common good and interest in individual health benefits emerged. Also, the value attributed to genetic research results was filtered through the concept of utility: clinically valid or actionable results, valuable because useful vs. research results, irrelevant. These findings align with previous literature, which showed that reciprocity, solidarity, altruism, and utility were important elements in the participant-study relationship and the meaning attributed to genetic research results and disclosure [17–20].

A previous study with CHRIS participants on return of secondary findings showed that participants wished to make autonomous choices about communication and disclosure according to a series of criteria that were important for them to make such a decision [21]. Based on this, the return of result policy of the CHRIS study was refined. With the new policy, participants are provided a model of four sample diseases caused by genetic variants, which differ in risk and opportunity for prevention and treatment [22]. In the informed consent, participants are asked to express their preferences for recontacting for each type of genetic variant shown in the model. These choices can be changed over time. Hence, a dynamic informed consent model proved crucial for adequately responding to participant needs and addressing the heterogeneity of participant preferences for disclosure.

In this study, respondents expressed different views on what was acceptable or desirable for disclosure when invited to participate in an RbG study. This depended on the perceived impact that such knowledge would have in their life (burden, awareness, possibility of prevention and treatment). This resulted in different preferences for disclosure and invitation in different situations. Previous literature reported on the importance of clinical validity and clinical utility in affecting biobank participants’ and patient cohorts’ preferences for disclosure [20, 23].

Based on our findings, policy on RbG approach may be improved by addressing the following points: (1) Participants should be made aware of the possibility of conducting this type of study beforehand. (2) Participants should be offered granular options for consent to participation in RbG studies, and the options should reflect participant preferences.

Empirical studies with different methodologies will allow an in-depth understanding of participant preferences and views, which will allow for further improve the informed consent and RbG policy.

The identified heterogeneity of participant preferences has broader research and practice implications. First, researchers should be attentive to what is important for participants and adequately address participant needs and views as partners. Second, as already highlighted in other empirical studies [19, 20, 23–25], the context in RbG is essential, both in a sense previously identified [7] and at a societal level. Considering the specific disease under study and the meanings attached to it (e.g., possible stigma or discrimination) and the socio-economic conditions (including the access to health care) where the study is conducted would allow a deepened reflection on the societal implications of RbG studies and the extent to which specific research practices are transferrable to other contexts. This implies a limitation in the applicability of the present study’s findings in other contexts.

The embedded design allowed us to elicit perspectives grounded in the experience of participating in an RbG study. The mixed-method research approach was relevant to the research aims. With the analytical strategy that informed the interpretation of the data, the qualitative results complemented the limitations of the survey results by showing a range of nuances beyond clear-cut numbers and allowing us to understand aspects where the quantitative part became less informative. Given the sample size, our work does not aim to be representative of the CHRIS cohort.

As the analysis was conducted on an English translation, we established measures to ensure accuracy, such as proofreading the translation and comparing independent coding of the German original data and the English version. The results were also thoroughly discussed with one of the authors who conducted the interviews, thus further assessing their adherence to the collected data, meaning, and interpretation.

## Conclusion

This study allowed us to identify crucial aspects of participant perspective and response to the RbG research approach. In line with existing recommendations [6, 7, 18, 20, 23, 25], this study highlighted the importance of a suitable communication strategy and adequate original informed consent to address the specificities of the RbG research approach. Co-design processes will be crucial for identifying solutions.

The communication strategy and the informed consent model chosen by a research study are key for aptly responding to approaches that are increasingly widely used in genomics and genetics research, such as RbG approaches.

### Supplementary information

Supplementary Information

## Data Availability

The datasets generated and analyzed during the current study are available in the CHRIS study repository. For an application for CHRIS data, contact the CHRIS study access committee (access.request.biomedicine@eurac.edu). Further information on the CHRIS study is available at https://www.eurac.edu/en/institutes-centers/institute-for-biomedicine and https://it.chris.eurac.edu/.
