# Supplementary material for: Participant perspective on the recall-by-genotype research approach: a mixed-method embedded study with participants of the CHRIS study
Source: Eur J Hum Genet. 2023 Jan 4;31(11):1218–27. doi: 10.1038/s41431-022-01277-6 (PMC10620385; doi:10.1038/s41431-022-01277-6)
Supplement: Supplementary file 1 — Supplementary Information [file 41431_2022_1277_MOESM1_ESM.docx]

**Supplementary Information 1: Questionnaires and interview guide.**

The first questionnaire, which focused on the emotions at the time of invitation and satisfaction with the information received, was submitted before the clinical examination. The interview covered reasons for participation, response to invitation and assessment of the information provided, views, preferences, and concerns on disclosures, return of research results, and participation in future RbG studies. After the interview, participants answered the second questionnaire, which was focused on both the current RbG experience (emotions and concerns related to the disclosed information, preferences for disclosure of carrier status and return of individual genetic research results, satisfaction with the provided information) and preferences for recruitment and disclosure in further hypothetical studies (interest in further participation, evaluation of RbG research practices, preferences for disclosure of disease under study). The double assessment of emotions was meant to capture possible changes following participation. The questionnaires and the interview guide were informed by the insights obtained from a previously conducted qualitative study on the return of research results (21). The questionnaires and the interview guide are reported below.

## Questionnaire 1

Dear PAREGEN in-depth neurological examination participant, thank you for participating in our study today! Before you start the examinations, we would like to know how you felt when you received the invitation. All data will be treated confidentially and used to improve our communication process.

1. How did you feel when you received the invitation for today’s study? Please choose a value between 1 (I did not feel) and 5 (I felt strongly) for each of the following emotions.

When I received the invitation, I felt:

Curious

Anxious

Worried

Delighted

Upset

Carefree

Relieved

Nervous

Other:_

1.2. Why did you feel this way? Please briefly describe your personal reflections. __

2. Did the information materials you received before your participation adequately answer your questions about the study?

No, many questions were not answered

No, some answers are still unclear

Yes, most of the answers were clarified

Yes, all the questions were answered

Thank you for your feedback.

## Questionnaire 2

Dear participant in the in-depth neurological examination PAREGEN,

Your opinion about today’s study is important for us!

Through this questionnaire, we would like to collect your evaluation of the study and the information received. All data will be treated confidentially and will be used to improve our communication process.

1. How do you feel **after** participating in the study?

Please choose a value between 1 (I did not feel) and 5 (I felt strongly) for each emotion mentioned.

After participating in the study, I feel:

Curious

Anxious

Worried

Delighted

Upset

Carefree

Relieved

Nervous

Other:__

1.2. Why do you feel this way? Please briefly describe your reflections. __

2. Did you think about whether you have the gene variant associated with Parkinson's disease when you received the invitation for today's study?

Yes

No

3. Are you concerned that you may be a carrier of the Parkin gene variant?

Very concerned

Concerned to some degree

A little concerned

Not at all concerned

I don’t know

4. How would you assess your risk of developing Parkinson's disease yourself at some point?

Very high risk

Increased risk

Low risk

No risk

I don’t know

5. Please indicate to what extent you agree with the following statements:

1. I estimate my risk of developing Parkinson's disease to be higher today than before I was invited to participate in this study.
2. I am more worried about developing Parkinson's myself today than I was before I was invited to take part in this study.

Disagree

Rather not agree

Neither agree nor disagree

Rather agree

Agree

6. Would you like to have known whether you are a carrier of the gene variant or whether you belong to the control group without the gene mutation?

Yes

No

7. How likely do you think you belong to the control group that does not carry the Parkin gene variant?

Very likely

Rather likely

Neither likely nor unlikely

Rather unlikely

Very unlikely

8. Do you have relatives who have Parkinson's disease?

Yes

No

9. As part of this study, you will receive the clinical findings of your neurological examination if they are relevant to your health. However, it is not intended that you will receive genetic research results (either individual or general) from this study. What do you think about that?

That is fine with me.

I think that's a pity.

I don’t care.

10. Did the on-site information and advice address and answered all your questions?

No, many questions were not answered

No, some answers are still unclear

Yes, most of the answers were clarified

Yes, all questions were answered

11. Were possible questions or doubts sufficiently answered or clarified during your participation?

Yes

No

If no: Which questions could not be answered? Why could you not be answered?__

12. In the future, we plan to conduct further studies of this kind.

a) Would you like to participate in similar studies?

Yes

No

If No: What conditions would have to be met for you to participate again?__

b) Would you have preferred that we had not invited you to today's study?

If yes: Why? Please briefly describe your motivations._

13. Some other research institutes do not inform their study participants of the genetic disease associated with the investigated gene variant. When they invite participants to participate in follow-up studies, they simply refer to "further genetic research."

a) How do you evaluate this procedure?

Very negative

Rather negative

Partly negative/partly positive

Rather positive

Very positive

b) If you were invited to participate in another study, would you want to know to which disease the investigated gene variant is associated?

Yes

No

This is not important to me.

c) Gene variants may be associated with a wide variety of diseases. Due to their mild symptoms, these can be diseases that have only a minor impact on everyday life. However, severe diseases for which there is no cure may also be genetically determined.

Would the type of disease influence your decision whether you want to know what disease it is?

Yes

No

Thank you for your feedback.

## Interview

**Expectations**

Why are you participating in today's study?

What were your expectations?

**Response to invitation**

Did you understand why you were selected to participate in this study?

What did you think when you received the invitation?

Did you think about your health?

How did these thoughts make you feel? / How did you feel?

**Communication *Parkin* gene variant**

Did you think about whether you have the gene variant associated with Parkinson's disease?

Would you like to know which group you belong to? Why?

What feelings does the thought that you might have the gene variant trigger in you?

How do you estimate your risk of developing Parkinson's disease at some point?

Do you assess your risk differently today than before participating in this study?

Are you concerned that you may carrythe gene variant? Why?

If you had not been invited to participate in this study, you probably would not have thought about it:

Would you have preferred not to have been invited?

**Results**

You are not expected to receive research results from this study - neither individual nor general:

What do you think about it?

**Communication**

We would also like to know how you rate this study’s communication.

Did the information you received before participation leave any doubts or questions you would have liked to have clarified before the study?

Are there any doubts or questions you would like to ask now?

Do you think a follow-up is necessary or desirable?

**Further studies**

In the future, we plan to conduct further studies of this kind.

Would you participate in other similar studies? Why?

Would you have preferred not to be invited to this study?

Some similar studies do not tell their participants which disease the genetic variant being studied is associated with. What do you think about this? Would this be a good solution?

Would you like to know to which disease the genetic variation being studied is related? Why/why not?

Do you think it makes a difference which disease it is? (fatal disease vs. mild disease)
